# Top-down guidance of attention to food cues is enhanced in individuals with overweight/obesity and predicts change in weight at one-year follow up

**DOI:** 10.1038/s41366-018-0246-3

**Published:** 2018-11-21

**Authors:** Panagiota Kaisari, Sudhesh Kumar, John Hattersley, Colin T. Dourish, Pia Rotshtein, Suzanne Higgs

**Affiliations:** 1 School of Psychology, University of Birmingham, Edgbaston, Birmingham, B15 2TT UK; 2 Warwick Medical School, University of Warwick, Coventry, UK; 3 Warwickshire Institute for the Study of Diabetes, Endocrinology and Metabolism (WISDEM), University Hospitals of Coventry and Warwickshire (UHCW) NHS Trust, Coventry, UK; 4 Human Metabolic Research Unit, University Hospitals Coventry and Warwickshire NHS Trust, Coventry, UK; 5 P1vital, Wallingford, Oxfordshire, OX10 8BA UK

**Keywords:** Risk factors, Medical research

## Abstract

**Background:**

Changing eating behaviour may be challenging for individuals with obesity and this may be related to attentional bias towards food. Previous paradigms used to assess attentional bias to food stimuli have not distinguished between bottom-up processes related to assessment of rewarding stimuli versus top-down processes related to effects of mind-set on attention. We investigated whether attentional bias for food cues varies between individuals with overweight/obesity and healthy weight individuals, due to differential top-down control of attention. We also determined whether top-down biases predict food consumption in the lab and weight change in our sample over one-year.

**Methods:**

Forty-three participants with overweight/obesity and 49 healthy weight participants between the ages of 18 and 58 participated. Participants completed two attention tasks in a counterbalanced order: (i) a priming task assessing bottom-up control of attention and (ii) a working memory task assessing top-down control of attention. Eating behaviour was assessed by a taste test. At one-year follow-up participants returned to the laboratory to assess changes in their body mass index (BMI).

**Results:**

The healthy weight and overweight/obese groups did not differ in demographics and baseline measures (appetite, food liking, taste test food intake). Participants with overweight/obesity showed greater top-down attentional bias towards food cues than did healthy weight participants but had no difference in bottom-up attentional bias. Top down attentional bias towards food cues predicted weight change over one-year but did not predict food intake in the taste test.

**Conclusions:**

The present findings illustrate that the relationship between attentional bias for food, food intake, and body weight is complex. Top-down effects of mind-set on attention, rather than bottom-up control of attention to food may contribute to patterns of eating that result in development and/or maintenance of overweight/obesity. Interventions targeted at top down biases could be effective in facilitating prevention of weight gain.

## Introduction

Changes in the food environment such as the abundant presence of food cues have been proposed to play a role in the increased prevalence of obesity [1, 2]. Individual differences have been reported in responsiveness to food cues, and individuals with a high food-cue responsiveness (attentional bias), may be more vulnerable to overeating and weight gain [3]. Understanding why some people find it more difficult to ignore food cues in the environment than do  others could be helpful in designing personalised and more effective weight management interventions.

Two mechanisms have been proposed to explain why motivational objects, such as food cues attract attention. The incentive-sensitisation theory proposes that food attracts attention due to its rewarding properties in a bottom-up manner by conditioning. Thus, after repeated associations between food cues and a rewarding experience, the cues become salient and attract attention [4].

An alternative mechanism was  suggested by Cox et al. [5] who proposed a motivational framework. These authors suggested that preoccupation with an issue, such as alcohol consumption, leads to biases for related information. Recent evidence suggests that thinking about food (maintaining food cues in working memory) biases attention towards food [6–8]. These data suggest that food-related attentional biases are mediated, at least partly, through top-down processes (driven by endogenous attention) related to mind-set, as well as through bottom-up conditioning processes.

Investigation of attentional processing of food cues in individuals with overweight/obesity has yielded mixed findings [9–14]. Indeed, in a review of the literature, Field et al. [15]. concluded that there is weak evidence that obesity is associated with an enhanced attentional bias toward food cues. Similarly, while some studies have found that individual differences in attentional bias to food cues are positively related to food intake [16, 17], others have failed to identify any significant relationships [12, 13]. This may in part be because the factors that contribute to biased processing of food cues are likely to be many and varied and the causal relationships are unclear (e.g. see work by Tapper and colleagues [18]). In fact, only two studies to date have investigated a causal association between attentional bias to food and weight gain. Calitri et al. [19], reported that cognitive biases predicted changes in BMI over one-year and Yokum et al. [20]. reported that attentional bias to food was associated with increased risk for weight gain in a sample of adolescent girls.

Previous paradigms used to assess attentional bias to food (e.g. Stroop, dot probe task) do not distinguish between top-down versus bottom-up attentional processes, which may explain why it has been reported that different measures of attentional bias for food do not always correlate with one another (e.g. [21]). However, food preoccupations have been reported to be predominant among heavier individuals [22] and a memory bias for food stimuli has been described in individuals with obesity [10]. This suggests that individuals with obesity or those prone to gaining weight may show exaggerated top-down bias of attention to food cues because they are more likely to retain food-related thoughts in mind and/or find it easier to elaborate food thoughts in working memory than are healthy weight individuals.

Our aim was to investigate attentional biases for food cues in both healthy weight individuals and individuals with overweight/obesity using a paradigm that assesses both bottom-up (exogenous) and top-down (endogenous) guidance of attention. In one version of the task we assess attention to food cues when participants are asked to hold food-related information in mind (top down condition) and we compare this with a condition in which participants are merely exposed to a food cue but not asked to keep it in mind (bottom up condition). An additional aim was to investigate whether attentional bias predicts eating behaviour in a laboratory setting, and weight change after one-year in our sample. We hypothesised that individuals with overweight/obesity might display both greater bottom-up and top-down attentional biases to food-related stimuli but that the effect might be stronger for the top-down attentional guidance. We further hypothesised that these attentional biases would predict both food intake in the lab and weight change over one-year in the whole sample.

## Materials and methods

### Participants

Men and women, aged 18–60 years, were recruited through posters, emails and mailshots. Based on previous results reported from Werthmann et al. [13], it was calculated that there would be 80% power to detect a difference of ~28% in food intake at the 5% significance level with 88 participants. BMI was verified at study enrolment for study group classification. Subjects with 18.5 < BMI < 25 were classified as healthy weight (HW), and subjects with BMI ≥ 25 were classified as overweight/obese (OW/OB). To reduce demand characteristics, the study was advertised as research examining eating habits and memory function. Participants were required to be fluent English speakers, as we had no resources for translators and to have a BMI between 18.5 and 40. Exclusion criteria were: (i) the presence or a history of a diagnosed eating disorder, a psychiatric, neurological or medical illness including diabetes, (ii) the presence or a history of tobacco use, drug abuse or the use of any medication, within the past month, that might influence eating behaviour and/or body weight, (iii) the presence of a food intolerance and/or allergy. Participants could choose to take part in exchange for money (50 UK pounds) or course credits. One hundred and five participants took part in the study. Thirteen participants were excluded: (i) six because they were classified as underweight, (ii) five due to a high error rate on the attention tasks [>3 SD from the mean], and (iii) two as outliers for food intake (>2 SD from their group mean). The final sample comprised 92 participants; 49 HW and 43 OW/OB participants (18 individuals with obesity and 25 individuals with overweight). See Table 1 for details of the participant characteristics.

**Table 1:** Baseline characteristics of groups

	Lean (*n* = 49)		Overweight/obese (*n* = 43)		*t*-test/*x*^2^	
**Demographics**	**Mean (SD)**	**Min**-** Max**	**Mean (SD)**	**Min-Max**	***t*** **(90)**	***p***
Age (years)	27.27 (9.95)	18–53	31.16 (9.82)	18–58	−1.89	0.06
	***n*** **(%)**		***n*** **(%)**		***x*** ^**2**^ **(1)**	***p***
Gender (% female)	43 (87.8)		26 (60.5)		9.10	<0.01
**Ethnic background**	***n*** **(%)**		***n*** **(%)**		***x*** ^**2**^ **(3)**	***p***
White/White British	30 (61.2)		23 (53.5)		2.48	0.48
Asian/Asian British	15 (30.6)		12 (27.9)			
Black/African/Caribbean/Black British	3 (6.1)		7 (16.3)			
Mixed/multiple ethnic groups	1 (2.0)		1 (2.3)			
**Body composition**	**Mean (SD)**	**Min-Max**	**Mean (SD)**	**Min–Max**	***t*** **(51)**	***p***
BMI (kg/m^2^)	21.46 (1.58)	18.7–24.9	30.46 (4.51)	25.0–42.8	−12.43	<0.001
					***t*** **(71)**	
Percent body fat (%)	24.25 (6.72)	7.3–37.1	35.2 (10.26)	16.3–51.9	−5.98	<0.001
					***t*** **(57)**	
Lean weight (kg)	45.10 (6.04)	35.2–62.1	56.62 (13.28)	34.4–90.0	−5.24	<0.001
					***t*** **(54)**	
Resting metabolic rate	1395.43 (152.13)	1099–1781	1775.10 (354.32)	1195–2694	−6.45	<0.001
**Physical activity**	**Mean (SD)**	**Min-Max**	**Mean (SD)**	**Min−Max**	***t*** **(90)**	***p***
MET—min/week	2617.55 (2692.25)	0–13332	2815.76 (2550.63)	0–11688	−0.36	0.72
**Impulsivity**	**Mean (SD)**	**Min-Max**	**Mean (SD)**	**Min−Max**	**t(90)**	**p**
BIS—total	63.12 (10.50)	47–97	62.25 (8.02)	45–83	0.44	0.66
**Appetitive drive to consume highly palatable food**	**Mean (SD)**	**Min-Max**	**Mean (SD)**	**Min−Max**	***t*** **(90)**	***p***
PFS—total	2.74 (0.81)	1.4–4.3	2.81 (0.78)	1.4–4.4	−0.38	0.71
					**MANOVA**	
					**Between-groups effects**	
**Eating behaviour**	**Mean (SD)**	**Min-Max**	**Mean (SD)**	**Min–Max**	***F*** **(1,90)** ***ηp*** ^**2**^	***p***
TFEQ—restraint	8.45 (4.84)	1–19	9.72 (4.33)	1–18	1.74 0.02	0.19
TFEQ—disinhibition	6.47 (3.31)	1–14	7.81 (3.35)	1–14	3.73 0.04	0.06
TFEQ—hunger	5.69 (3.14)	1–12	4.88 (2.70)	0–12	1.73 0.02	0.19
DEBQ—dietary restraint	2.48 (0.87)	0.9–4.2	2.87 (0.73)	1.5–4.5	5.25 0.06	0.02
DEBQ—emotional eating	2.21 (0.95)	0.9–4.9	2.22 (1.02)	0.7–4.3	0.001 0.00	0.98
DEBQ—external eating	3.12 (0.63)	1.8–4.5	3.08 (0.61)	2.0–4.5	0.07 0.00	0.79
**Psychological distress**	**Mean (SD)**	**Min-Max**	**Mean (SD)**	**Min–Max**	***F*** **(1,90)** ***ηp*** ^**2**^	***p***
HADS—anxiety	7.37 (4.05)	1–16	6.16 (3.68)	0–15	2.20 0.02	0.14
HADS—depression	3.45 (2.81)	0–12	3.70 (2.86)	0–12	0.18 0.00	0.68

Change in body weight data at 12 months were available for 70 participants; 36 in the HW group and 34 in the OW/OB group; 52 women and 18 men. Thirteen participants provided self-report BMI data

All participants provided written informed consent. The study was approved by the National Research Ethics Service (NRES), NRES Committee West Midlands—The Black Country and the University of Birmingham Research Ethics Committee.

### Measures

#### Self-report measures

*The Three Factor Eating Questionnaire (TFEQ)* [23], a 51-item questionnaire was used to assess “cognitive restraint of eating”, “disinhibition” and “hunger”. In the present study the Cronbach’s alpha was 0.83, 0.76 and 0.75 for the three subscales, respectively.

*The Dutch Eating Behaviour Questionnaire (DEBQ)* [24], a 33-item questionnaire was used to assess “emotional eating”, “external eating” and “dietary restraint”. In the present study, the Cronbach’s alpha was 0.94, 0.83 and 0.91 for the three subscales, respectively.

*The Power of Food Scale (PFS)* [25], a 15-item questionnaire was used to assess the appetitive drive to consume highly palatable food. In the present study, the total score was used and the Cronbach’s alpha was 0.91.

*The Hospital Anxiety and Depression Scale (HADS)* [26], a 14-item questionnaire was used to assess anxiety and depression. The Cronbach’s alpha in the present study was 0.83 and 0.71 for the Anxiety and Depression subscales, respectively.

*International Physical Activity Questionnaire*—Short Version (IPAQ-SF) [27], a 7-item questionnaire was used to assess physical activity during the last 7 days. Computation of the total score for the short form requires summation of the duration (in minutes) and frequency (days) of walking, moderate-intensity and vigorous-intensity activities.

*Barratt Impulsivity Scale* *(BIS)* [28], a 30-item questionnaire was used to assess impulsivity. In the present study, the total score was used and the Cronbach’s alpha was 0.80.

#### Attentional tasks

We used an identical experiment and procedure to that reported previously by our group [6] that was designed to allow separate assessment of both bottom up, i.e. automatic attentional processing of exogenous cues and top down guidance of attention that relies on endogenous processes, such as having a particular mind-set. In brief, participants were asked to complete a selective attention task in two contexts: a priming task that assessed the involvement of bottom-up attention capture; and a working memory task that assessed the contribution of top-down attentional biases. The two tasks were very similar, but differed in the instructions to the participants. In both contexts, the selective attention task was identical. Participants were asked to identify the side of the screen in which a target stimulus (circle) appeared. The distractor stimulus was a square. In the search array both the target and distractor were presented simultaneously with two pictorial stimuli, which were irrelevant to the selection task. The priming and working memory tasks differed in the context in which the selective attention was assessed. In the bottom-up priming task, participants were asked to identify a cue (e.g. pictorial image) but not to hold it in memory. In the top-down working memory task, participants were asked to hold a cue in memory across the trial (the selective attention task) in order for it to be matched in a subsequent memory test. The relation between the cue and pictorial images at the search array defined three conditions: valid, neutral and invalid. On valid trials, the target was next to an image that was the same as the cue and the distractor was next to an image from a different semantic category. On invalid trials, the distractor was flanked by an image that was the same as the cue and the target was flanked by an image from one of the other cue categories. On neutral trials, both the target and distractor were flanked by images from categories different from the cue. Valid, neutral and invalid trials occurred randomly with equal probability. The task context (priming, working memory) was manipulated as blocks, with 650 trials in each. The stimuli included 10 pictures from three categories: food (e.g. Apple, pizza), office stationary (e.g. sellotape, pencil) and household items (e.g. Spanner, bucket). An effort was made to control for visual complexity and matched stimuli on various visual characteristics when selecting the food and non-food objects. Each pictorial image presented a single achromatic object on a black background and was 480 × 480 pixels in size. The same images were used as cues and were displayed in the search array.

##### Trial sequence

A trial started with a central fixation cross for 600 ms, followed by a cue for 500 ms. After the cue, a fixation cross appeared for 200–1000 ms (randomly chosen), followed by the search array, which consisted of a target (a circle) and a distractor (a square) that appeared randomly to the left or right of fixation. Participants had to press ‘c’ if the circle appeared on the left and ‘m’ if it appeared on the right side of the screen. The target and the distractor were presented next to two images taken from different categories (e.g. food, office, household items). The inter-trial interval was 400 ms. In the working memory task, 20% of the trials ended with a memory probe that followed the search display to check that the participants were performing the task correctly and had remembered the cue as instructed. On the memory probe trials, an item from the same category as the cue appeared for 3000 ms and the participants indicated whether the item was the same or different to the cue. Participants pressed ‘c’ if the item matched the cue or ‘m’ if it was different. In the priming bottom-up task the cue disappeared after 250 ms on 20% of the trials and a different image appeared in its place. On these trials, participants were required to withhold their response to the search task.

##### Taste test

Food consumption was measured by means of a bogus taste test [29]. During the taste test, participants were instructed to rate bowls of high-energy foods (≈255 g of chocolate (529 kcal/100 g), ≈150 g of chocolate cookies (502 kcal/100 g), ≈50 g of crisps (526 kcal/100 g), ≈130 g of salted biscuits (516 kcal/100 g)) and bowls of low-energy foods fruit and vegetables (≈50 g of nectarine (45 kcal/100 g), ≈250 g of melon (24 kcal/100 g), ≈250 g of cherry tomatoes (20 kcal/100 g) and ≈225 g of cucumber (10 kcal/100 g)) in terms of their visual attractiveness, smell and taste. All food items were purchased from Sainsbury’s UK. Participants were instructed to taste and rate the foods in a particular order, as consumption order could affect the taste ratings. Each participant was given 30 min to complete their ratings and informed that after finishing their ratings they were free to eat as much of the foods as they liked, as they were not going to be used for other participants. A glass of water was provided. Consumption was determined by the difference in weight of foods from pre-assessment to post-assessment. Participants were unaware that their food intake was weighed.

### Procedure

Testing took place between 09:00 a.m. and 10:15 a.m., and participants were asked to arrive at the Unit with instructions not to consume any food for 9–10 h prior to their arrival. Participants were also advised to avoid exercising on the day of testing. Participants were asked to report on demographic characteristics and rate their baseline hunger, fullness, desire to eat and thirst on 100 mm Visual Analogue Scales (VAS) anchored by word descriptions at each end that express two extreme states of the condition (e.g. “Not Hungry at all”, “Very Hungry”). Resting energy expenditure (REE) and body composition measurements were also made using a metabolic cart (Metalyzer 3B, Cortex, Germany) and a BodPod using air displacement plethysmography (Cosmed, Rome, Italy) [30] or a TANITA Body Fat Scale using advanced bioelectrical impedance analysis technology, respectively.

Since motivational state is known to affect cognitive biases to food [31], prior to the attentional task, participants consumed a meal consisting of a cheese sandwich on white bread and a glass of orange juice (energy content = 500 kcal). After a break of 20 min, appetite ratings were scored again by VAS. Participants were then asked to complete the bottom-up and top-down tasks, with an option of a 5 min break between tasks. The bottom-up and top-down tasks were completed in a counterbalanced order. After completion of these tasks, appetite ratings were scored again by VAS. Subsequently, a bogus taste test was performed. After the taste test, the participants completed another set of VAS, and finally were left alone in a room to complete the questionnaires on eating behaviour, physical activity, impulsivity and psychological distress. At the end of the experimental session participants were thanked for their time, and reimbursed for participation.

In the 1-year follow up, participants were asked to attend a brief session during which body composition measurements were obtained with a BodPod or a TANITA Body Fat Scale. Participants who were not able or willing to attend the follow-up session were asked to provide a self-report measure of body weight. As body weight measurements may vary depending on fasting state, all participants were instructed to avoid eating and drinking for 9–10 h before the body weight assessment.

### Analysis

To control study-wise Type I error rate, comparisons of eating measures, personality, and food liking scores between groups were conducted initially using multivariate analysis of variance (MANOVA) and only followed up by univariate ANOVAs when the MANOVA was significant. Changes in ratings of hunger, desire to eat, fullness and thirstiness VAS scales were analysed using mixed ANOVAs, with time as a repeated measure and BMI status as a between subject measure. One-way ANCOVA with BMI status as a factor and gender and restrained eating as covariates was used to compare food intake (in kcal) between groups.

Incorrect responses to the search task, memory task, and catch trials, as well as reaction times (RTs) that were ±3 standard deviations from the mean were removed. Differences in RTs between tasks (top-down, bottom-up), trials (valid, neutral, invalid), and cues (food vs. non-food items) were analysed using mixed ANOVAs with the task condition as a repeated-measure and group (HW, OW/OB) as a between-subjects factor. Post-hoc tests were corrected using Bonferroni correction.

A regression analysis was conducted to examine whether attentional bias predicts food intake and BMI change. Given that, age, gender, physical activity levels, body composition measurements, eating styles, and impulsivity are all possible determinants of food intake and BMI change [32–35], these factors were included as covariates. Baseline levels of appetite (after the satiety manipulation) and food liking were included in the food intake model [29]. Inspection of variance inflation factors did not reveal an issue with multicollinearity (all VIF < 6) although gender and weight were moderately correlated.

## Results

### Food intake

The mean total food intake was 386 kcal (SD = 180.87) for the HW and 473 kcal (SD = 250.64) for the OW/OB group. The energy intake from high-energy food items was 312 kcal (SD = 169.97) for the HW and 412 kcal (SD = 255.19) for the OW/OB group, and the energy intake from fruit and vegetables was 74 kcal (SD = 49.44) and 61 kcal (SD = 46.60) for the two groups, respectively. Controlling for gender and dietary restraint, no group differences were observed in total intake, *F*(1,88) = 1.26, *p* = 0.27, *ηp*^2^ = 0.01, intake of high-energy food items, *F*(1,88) = 2.28, *p* = 0.14, *ηp*^2^ = 0.03 or intake of  fruits and vegetables, *F*(1,88) = 2.78, *p* = 0.10, *ηp*^2^ = 0.03.

### Appetite and liking ratings

There was a main effect of group upon the hunger ratings *F*(1,90) = 4.57; *p* = 0.04, *np*^2^ = 0.05, and a trend for a significant effect upon the desire to eat ratings *F*(1,90) = 3.68; *p* = 0.06, *np*^2^ = 0.04, with the HW group scoring on average higher on both those measures of appetite than the OW/OB group. There was no main effect of group upon the fullness *F*(1,90) = 1.09; *p* = 0.30, *np*^2^ = 0.01, and thirstiness ratings *F*(1,90) = 0.49; *p* = 0.49, *np*^2^ = 0.01. No significant interactions were found (all *p*s > 0.05) (Table 2).

**Table 2:** Mean subjective appetite (hunger, fullness, desire to eat, thirstiness) by group

	Lean (*n* = 49)				Overweight/obese (*n* = 43)				Repeated measures ANOVA	
Appetite ratings	Before meal	After meal	Before taste test	After taste test	Before Meal	After meal	Before taste test	After taste test	Between-groups effects	
	Mean (SD)				Mean (SD)				*F* (1,90) *ηp*^2^	*p*
Hunger	62.18 (24.39)	23.86 (20.73)	43.25 (24.26)	11.49 (15.77)	50.42 (26.56)	19.54 (19.26)	31.98 (25.08)	10.21 (17.83)	4.57 0.05	0.04
Fullness	19.80 (22.32)	61.06 (22.90)	44.08 (22.71)	80.28 (19.76)	23.44 (18.29)	64.02 (23.39)	49.00 (26.50)	82.98 (16.80)	1.09 0.01	0.30
Desire to eat	67.74 (21.31)	30.47 (22.99)	49.16 (22.95)	15.43 (16.03)	56.09 (24.66)	26.09 (20.48)	42.61 (27.34)	12.67 (13.07)	3.68 0.04	0.06
Thirstiness	77.20 (22.60)	42.94 (24.65)	67.27 (25.71)	28.04 (24.02)	73.07 (25.36)	41.44 (28.14)	61.02 (25.22)	28.86 (23.46)	0.49 0.01	0.49

No effect of group was observed upon the liking ratings of the high energy foods, *F*(4,84) = 6.71, *p* = 0.61; Wilk’s Λ = 0.97, *ηp*^2^ = 0.03, or ratings of fruits and vegetables *F*(4,85) = 1.39, *p* = 0.25; Wilk’s Λ = 0.94, *ηp*^2^ = 0.06 (data not shown).

### Attention task performance

In both tasks, search accuracy was high (92% correct for the bottom-up and 87% for the top-down). There was no evidence of a speed–accuracy trade off.

We tested our priori hypothesis about the specific effect of having obesity on bottom-up and top-down selective attention mechanisms, using independent *t*-tests to assess whether group affected the ability to disengage attention and for attention to be captured by food in the context of bottom-up and top-down tasks. RTs for valid trials were subtracted from RTs for invalid trials. Increased values on this score translate to increased effort to disengage from the cued item when it was a distractor and enhanced capture of attention by the cue. When the cue was a food item, the biasing effect was greater than when it was a non-food item for both the bottom-up (*t*(91) = 2.95; *p* < 0.01) and the top-down task (*t*(91) = 4.81; *p* < 0.001). The biasing effect was significantly greater in the top-down task than the bottom–up task, both for the food cues (*t*(91) = 5.25; *p* < 0.01) and the non-food cues (*t*(91) = 4.91; *p* < 0.01).

**Fig. 1:**
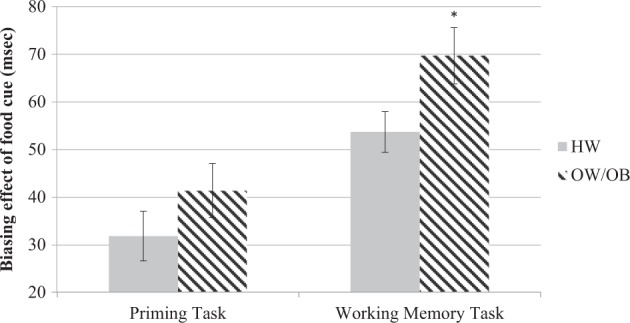
Biasing effect of food cue (=RTs for food invalid trials – RTs for food valid trials; in milliseconds), for the priming and working memory task by group. The group with OW/OB showed a significantly greater biasing effect for the food cues in the WM task indicating they were slower to disengage from food stimuli when holding food information in memory. *p < 0.05

The biasing effect for non-food cues did not differ between groups, for both the bottom-up (*F*(1,90) = 0.21, *p* = 0.65) and the top-down task (*F*(1,90) = 0.76, *p* = 0.39). However, the OW/OB group showed a significantly greater biasing effect for the food cues in the top-down task (*F*(1,90) = 4.96; *p* = 0.03, *ηp*^2^ = 0.05), while no difference was observed in the bottom-up task (*F*(1,90) = 1.56; *p* = 0.22) (see Fig. 1).

Replicating previous results, RTs were slower in the top-down than the bottom-up task (*F*(1,90 = 119.24; *p* < 0.001, *ηp*^2^ = 0.57) suggesting that the tasks differed in difficulty. RTs were faster for valid trials than the neutral and invalid trials, and were faster for neutral compared to invalid trials (main effect of validity: *F*(2,180) = 254.03; *p* < 0.001, *ηp*^2^ = 0.74). The validity effect was smaller for the bottom-up task (two-way interaction between task and validity (*F*(2,180) = 27.43; *p* < 0.01, *ηp*^2^ = 0.23). There was a main effect of cue (*F*(1,90) = 39.95; *p* < 0.001, *ηp*^2^ = 0.31) and a two-way interaction between task and cue (*F*(1,90) = 8.77; *p* = 0.004, *ηp*^2^ = 0.09). RTs were shorter on food cue trials in both the bottom-up (*p* = 0.01) and top-down tasks (*p* < 0.001), however the difference was smaller in the bottom-up task. All other interactions with group were non-significant (*p*_s_ > 0.05).

### Predictors of food intake

Age, gender, desire to eat ratings, food liking ratings, physical activity, body fat (kgs), lean weight (kgs), trait disinhibition and dietary restraint, PFS, impulsivity and the top-down attentional bias (=RTs for WM food invalid trials – RTs for WM food valid trials), were entered as predictors in the regression model. This model accounted for 40% of the variance in intake of high- energy food items. Ratings of desire to eat, physical activity, and impulsivity were the only significant predictors (see Table 3).

**Table 3:** Multiple regression model predicting food intake from highly palatable food items

Predictor	*B*	SE *B*	*β*
Age	−1.68	2.06	−0.08
Gender	17.12	91.52	0.03
VAS desire to eat (after offered meal)	2.61	0.99	0.26*
VAS liking	1.75	1.28	1.23
Physical activity (MET—min/week)	0.02	0.01	0.25**
Body fat (kgs)	3.93	2.04	0.22
Lean weight (kgs)	3.51	3.38	0.19
TFEQ—disinhibition	10.55	7.46	0.16
DEBQ—restraint	−12.05	25.27	−0.05
PFS—total	−50.17	30.60	−0.18
BIS—total	5.82	2.17	0.25**
Biasing effect of food cue held in WM	1.02	0.65	0.16
ANOVA	*F*(12,79) = 4.42**		
*R* ^2^	0.40		

*p < 0.05; **p < 0.01

### Weight change—one-year follow-up

Age, gender, levels of physical activity, body fat, lean mass, trait disinhibition and dietary restraint, PFS, impulsivity and the top-down attentional bias, as assessed at phase 1 of the study were entered as predictors in the regression model. This model accounted for 35% of the variance in BMI change. Gender, lean weight and top-down attentional bias were the only significant predictors (see Table 4).

**Table 4:** Multiple regression model predicting BMI change over a one-year period

Predictor	BMI change: 1 year follow-up		
	*B*	SE *B*	*β*
Age	0.02	0.01	0.15
Gender	2.14	0.59	0.88**
Physical activity (MET-minutes/week)	5.765E−5	0.00	0.14
Body fat (kgs)	−0.00	0.01	−0.04
Lean weight (kgs)	0.06	0.02	0.68**
TFEQ—disinhibition	0.07	0.04	0.21
DEBQ—restraint	−0.09	0.16	−0.07
PFS—total	−0.15	0.19	−0.11
BIS—total	−0.02	0.01	−0.16
Biasing effect of food cue held in WM	0.01	0.00	0.26*
ANOVA	*F* (10,59) = 3.16**		
*R* ^2^	0.35		

*p < 0.05; **p < 0.01

## Discussion

To measure attentional biases for food cues we used a paradigm that captures both bottom-up and top-down attentional processes. Both the HW and OW/OB groups demonstrated an attentional bias for food cues in the bottom-up task, suggesting that food cues bias attention more than non-food cues due to greater attractiveness of the cues [11]. In addition, holding food cues in mind biased attention towards food cues, as has been reported previously [7]. In line with our hypothesis, the OW/OB group were slower than the HW group to detect the target in invalid trials compared to valid trials when holding a food cue in working memory, indicating that individuals with overweight/obesity find it harder to disengage from food cues when food is in mind (top-down attentional bias).

The current results support the suggestion that mind-set is an important factor in modulating the expression of attentional biases to food stimuli [7, 15, 35]. Extending previous findings, the data suggest that (1) holding information in working memory affects responses to food-related cues in the environment and that (2) this latter effect is magnified in higher weight versus healthy weight participants. One possible explanation for the pattern of results observed is that higher weight people are more susceptible to the biasing effect of thinking about food on attention [36] due to their greater concerns and or preoccupations with food [22, 37].

For first time, we provide evidence that top-down attentional bias to food cues predicts weight change in our sample overall. To the best of our knowledge, only two other studies have investigated the predictive value of attentional biases in weight gain but these studies did not examine the specific contribution of top-down versus bottom-up biases [19, 20]. While impulsivity, physical activity and desire to eat ratings were significant predictors of intake, as has been found previously [38–40], we did not find that attentional biases for food cues predicted food intake in the taste test. Werthmann et al. [13] and Nijs et al. [12] also found no association between attentional biases to food and snack food intake. A recent analysis of intake during taste tests in the laboratory also failed to find a relationship between amounts consumed and BMI [29]. One possibility is that higher weight participants do not eat more than do their healthy weight counterparts because they may have self-presentation concerns that restrict food intake in the laboratory setting, especially where they are aware their food intake is being monitored. Nevertheless, these data suggest that further research is required to elucidate the complex relationships between top-down attentional biases, food intake and weight gain. Higher weight participants found it harder to disengage from food cues when holding food cues in memory. Therefore, it may be difficult for these individuals to ignore food cues in the environment, which could lead to overeating. One possibility that could be investigated is that holding food-related information in working memory affects the propensity to engage in opportunistic eating [41], and increase the frequency of eating episodes rather than promoting intake once an eating episode has been initiated (as assessed in the present study). Future research could test this hypothesis using the present design but incorporating an unanticipated opportunity to initiate an eating episode following the mandatory taste test [42].

We also found that gender and lean mass, but not body fat mass, were significant predictors of BMI change over a one-year period. Women gained more weight than men did  at one-year follow-up. However, gender was not equally distributed in our sample, and only 25.7% of participants at follow-up were men. Therefore, our finding that women are at increased risk for weight-gain should be interpreted with caution. Evidence suggests that fat-free mass plays a major role in appetite regulation, stimulating food intake, and our data contribute to this evidence base [43, 44].

As food cues are particularly prominent in the modern  food environment, understanding how individuals respond to these cues will be helpful in guiding tailored weight management programmes. Given the predictive value of top-down attentional bias to food cues on weight gain, it would be of great interest to examine the effects of interventions aimed at reducing food preoccupations on eating behaviours and attentional processes in people who show exaggerated attention to food cues.
